# Analysis of esophageal‐sparing treatment plans for patients with high‐grade esophagitis

**DOI:** 10.1120/jacmp.v14i4.4248

**Published:** 2013-07-08

**Authors:** Joshua S. Niedzielski, Jaques B. Bluett, Ryan T. Williamson, Zhongxing Liao, Daniel R. Gomez, Laurence E. Court

**Affiliations:** ^1^ Department of Radiation Physics The University of Texas MD Anderson Cancer Center Houston TX; ^2^ Graduate School of Biomedical Sciences The University of Texas Health Science Center at Houston Houston; ^3^ Department of Radiation Oncology The University of Texas MD Anderson Cancer Center Houston TX USA

**Keywords:** esophagitis, planning study, esophagus, radiotherapy, interfractional motion, partial sparing, toxicity

## Abstract

We retrospectively generated IMRT plans for 14 NSCLC patients who had experienced grade 2 or 3 esophagitis (CTCAE version 3.0). We generated 11‐beam and reduced esophagus dose plan types to compare changes in the volume and length of esophagus receiving doses of 50, 55, 60, 65, and 70 Gy. Changes in planning target volume (PTV) dose coverage were also compared. If necessary, plans were renormalized to restore 95% PTV coverage. The critical organ doses examined were mean lung dose, mean heart dose, and volume of spinal cord receiving 50 Gy. The effect of interfractional motion was determined by applying a three‐dimensional rigid shift to the dose grid. For the esophagus plan, the mean reduction in esophagus V50, V55, V60, V65, and V70 Gy was 2.8, 4.1, 5.9, 7.3, and 9.5 cm^3^, respectively, compared with the clinical plan. The mean reductions in LE50, LE55, LE60, LE65, and LE70 Gy were 2.0, 3.0, 3.8, 4.0, and 4.6 cm, respectively. The mean heart and lung dose decreased 3.0 Gy and 2.4 Gy, respectively. The mean decreases in 90% and 95% PTV coverage were 1.7 Gy and 2.8 Gy, respectively. The normalized plans’ mean reduction of esophagus V50, V55, V60, V65, and V70 Gy were 1.6, 2.0, 2.9, 3.9, and 5.5 cm^3^, respectively, compared with the clinical plans. The normalized plans’ mean reductions in LE50, LE55, LE60, LE65, and LE70 Gy were 4.9, 5.2, 5.4, 4.9, and 4.8 cm, respectively. The mean reduction in maximum esophagus dose with simulated interfractional motion was 3.0 Gy and 1.4 Gy for the clinical plan type and the esophagus plan type, respectively. In many cases, the esophagus dose can be greatly reduced while maintaining critical structure dose constraints. PTV coverage can be restored by increasing beam output, while still obtaining a dose reduction to the esophagus and maintaining dose constraints.

PACS number: 87.53 Tf

## I. INTRODUCTION

A common early side effect of external beam radiotherapy treatment for lung cancer is acute esophagitis. This toxicity can be expected, as treatment plans typically include some portion of the esophagus in the treatment field. Grade 3 esophagitis can occur in more than 20% of patients receiving three‐dimensional conformal radiotherapy, and as high as 44% for intensity‐modulated radiotherapy (IMRT) with concurrent chemotherapy (CCT) for lung cancer.^1^, ^2^, ^3^ This severe early effect can eventually lead to breaks in treatment, which can negatively affect long‐term survival.^(4)^ CCT can significantly increase tumors’ response to radiation and patients’ median survival duration, but may also increase the severity of early effects in normal tissue.^(5)^ Subsequently, early tissue toxicity can lead to late effects such as esophageal stricture, which can greatly affect quality of life.^(6)^

Past studies have examined the dosimetric quantities relevant to esophagus exposure and early toxicities.^3^, ^7^, ^8^, ^9^, ^10^, ^11^, ^12^, ^13^ A study by Zhou et al.^(14)^ showed that conventional DVH dosimetric techniques may not be sufficient to describe the 3D dose distribution of the esophagus. Previous studies have shown dosimetric variables that describe spatial distribution of dose, such as volume of esophagus receiving at least 20 Gy (V20) or length of esophagus receiving full circumference dose of at least 60 Gy (LE60), significantly predict high‐grade esophagitis.^12^, ^15^, ^16^, ^17^ Wei et al.^(3)^ found significance between V10–V45 and high‐grade radiation esophagitis (RE), and V20 and CCT as significant in multivariate analysis. Chapet et al.^(12)^ found significance between V40–V70 and grade 2 or higher RE. Other studies have shown V50 and V60 have a better correlation to patient outcome then other studies using DVH metrics.^(15)^ Since there is no single, definitive dosimetric value from these metrics that predicts RE, it is important to consider a variety of values.

While it is ideal to exclude the esophagus from the treatment field, this is not always possible. The concept of partial sparing of the esophagus has been studied previously.^7^, ^8^, ^9^ Even though a definitive relationship between volume or length of the esophagus receiving greater than a certain dose and sparing the esophagus has not been established, it is reasonable to surmise that length of esophagus receiving greater than a certain threshold dose induces esophagitis.^7^, ^8^, ^9^ Therefore, reduced esophageal dose plans may represent esophagus‐sparing treatment plans.

Interfractional motion can also contribute additional dose to the esophagus. A recent study by Cohen et al.^(18)^ showed a 24% incidence of interfractional esophageal motion greater than 5 mm. Therefore, interfractional motion should be considered to better represent esophageal anatomy under treatment conditions. Understanding the effect of simulated random motion on dosimetric quantities might show improved correlations between early normal tissue toxicity and dosimetric parameters.^(19)^

In this study, the effect of constraining the dose to the esophagus is examined using an automated radiotherapy treatment plan generation algorithm (mdaccAutoPlan). Two plans, an 11‐beam plan representative of a standard clinical IMRT plan and a second esophageal dose reduction plan, are compared with dosimetric quantities V50 to V70, as well as LE50 to LE70, in 5 Gy increments. Critical structure doses examined are: V50 of the spinal cord, mean lung dose, and mean heart dose. In addition, interfractional tumor motion was simulated and mean esophageal dose and LE60 were computed to examine the impact of using a reduced esophageal dose plan in place of a standard clinical plan. This inclusion of motion better represents the true anatomical conditions of dose to the esophagus.

## II. MATERIALS AND METHODS

In this planning study, 14 patients with NSCLC previously treated at MD Anderson Cancer Center were selected that presented with grade 2 or 3 acute esophagitis based on the Common Terminology Criteria for Adverse Events (CTCAE) version 3.0.^(20)^ These patients potentially benefit the most, in terms of early effects, from dose reduction to the esophagus. These patients’ 11‐beam clinical and reduced esophageal dose treatment plans were created in the Pinnacle treatment planning system (TPS) version 9.1 (Philips Radiation Oncology Systems, Fitchburg, WI) using an in‐house tool described below.

Each patient's original treatment plan was imported to a research Pinnacle server to generate two new treatment plans. This gives us the original treatment plan contours of the CTV, esophagus, lung, heart, and spinal cord. Using these structures will provide anatomical consistency with the original treatment plan and the algorithm generated plans. The first was an 11‐beam ‘clinical’ plan which resembles IMRT plans used for a current clinical trial comparing proton therapy to photons for lung cancer treatment at M.D. Anderson Cancer Center. The reduced esophageal dose plans also utilize 11 beams in the algorithm. Therefore, for comparison purposes, an 11‐beam clinical plan is advantageous. In addition, Zhang et al.^(21)^ reported clinicians preferred the 11‐beam mdaccAutoPlan for lung cancer IMRT than other lower beam number plans when overall plan quality, target coverage, lung sparing, and other normal tissue sparing was considered. The second plan type was a reduced esophageal dose plan designed to greatly reduce dose to the esophagus. Both treatment plan types were created using the mdaccAutoPlan radiotherapy plan generating algorithm, which is an in‐house tool that can be run from the Pinnacle TPS. Since there can be discrepancies in plan creation by a human treatment planner, the mdaccAutoPlan algorithm was selected to provide consistency between the generation of the two plan types.^(21)^

The following briefly summarizes the process by which the mdaccAutoPlan algorithm is used to generate 11‐beam clinical and reduced esophageal dose radiotherapy plans. Any structures to be analyzed are loaded into the patient plan. The 11‐beam clinical plan is then created utilizing a built‐in script. The algorithm creates a collection of structures, mainly for beam avoidance, and creates initial objective function parameters. For the objective function parameters, some structures, such as the heart, use maximum DVH dose as a constraint, while others, such as lung, utilize maximum equivalent uniform dose parameters. The algorithm starts with 19 treatment beams initialized from locations derived from a database of previously used treatment plans, and creates an IMRT plan. The algorithm then optimizes the beams by deleting the lowest weight beam, reanalyzing plan quality, adjusting beam geometry, and adjusting the objective function parameters until only 11 beams remain with high plan quality. The reduced esophageal dose plan follows the same workflow with an added objective constraint to the esophagus objective function parameter. For a more in‐depth discussion of mdaccAutoPlan, the reader is referred to Zhang et al.^(21)^

The original physician‐approved IMRT plans were not used in this study due to variation in size of PTV margins for different patients. Eleven‐beam clinical plans were created to deliver 37 fractions of 2 Gy for a total treatment dose of 74 Gy. The planning target volume (PTV) was defined as the clinical target volume plus uniform 5 mm margins for all treatment plans.

Two dosimetric quantities were studied for the esophagus between the two plans: the change in volume and the change in length of the esophagus receiving doses of 50, 55, 60, 65, and 70 Gy. In addition, critical structure doses, including the mean lung dose, volume of spinal cord receiving 50 Gy, and the mean heart dose, were investigated.

The mean lung and mean heart doses were automatically calculated in Pinnacle. The length of esophagus receiving a specific dose was calculated using the 50, 55, 60, 65, and 70 Gy isodose lines. If the isodose line completely covered the esophagus region of interest contour in the treatment plan, then the length of the esophagus was assumed to receive that dose for the entire computed tomography slice thickness.

Treatment quality was quantified by the 90% and 95% PTV coverage, and the changes between the two plan types were reported. Since 95% PTV is a constraint more often used clinically, it was used to determine the reduced esophageal dose plan's clinical suitability.

If the reduced esophageal dose plan's 95% PTV coverage dropped below that of the clinical plan, the esophagus plan was renormalized by increasing the plan's beam outputs until its 95% PTV coverage matched that of the clinical plan. After this normalization of the esophagus plan, the length and volume of esophagus receiving 50, 55, 60, 65, and 70 Gy were recalculated. In addition, the critical structure metrics were recalculated.

To understand the impact of interfractional motion on dosimetric variables between the 11‐beam clinical plan and the reduced esophageal dose plan, random motion was incorporated by shifting the dose grid with a three‐dimensional rigid shift. Similar methods have been applied assuming a Gaussian distribution of anatomical location over the course of treatment.^22^, ^23^ We applied a systemic and a random shift for all 37 fractions. Systemic shifts represent treatment planning setup uncertainties. Gaussian distributions with standard deviations of 3 mm and 5 mm were used to represent anatomical spatial distributions of structures on CT images. Calculated doses from each fraction were then summed to give the total treatment dose. This method was repeated 1000 times to obtain a distribution of delivered doses. The maximum esophageal dose and the LE60 values were compared between the two simulated plans for all 14 patients to examine any effect of interfractional motion on dosimetric quantities.

## III. RESULTS

**Figure 1:**
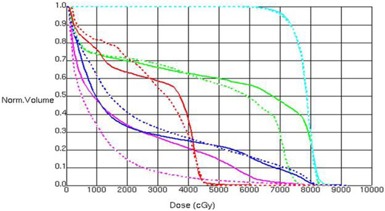
Example of DVH of 11‐beam plan (solid lines) and esophagus sparing plan (dashed lines) for a single patient. Structures displayed are: PTV (light blue), esophagus (green), spinal cord (red), lung (dark blue), and heart (pink).

**Figure 2:**
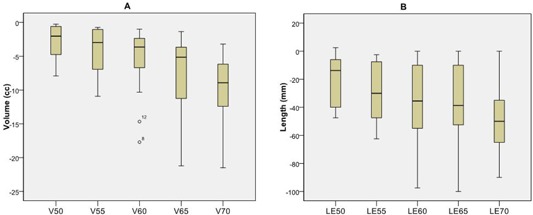
Box plot of change in dosimetric parameters between the 11‐beam clinical and reduced esophageal dose plans for all 14 patients. Change in esophageal volume (V; (a)) and in esophageal length (LE; (b)) receiving doses of 50, 55, 60, 65, and 70 Gy between the reduced esophagus dose plans and the 11‐beam clinical plans. Boxes= the 75th and 25th percentile; horizontal line= the mean value; whiskers= the range of data values; circles=outliers.

Dosimetric quantities were examined as the mean change from the 11‐beam clinical plan to the esophagus plan. There was, on average, a large reduction in dose to the esophagus when using the esophagus plan. An example of a dose‐volume histogram of the two plan types for a single patient is shown in Fig. 1. We can see qualitatively a large reduction in dose to the esophagus at higher doses, and little change in the PTV when using the reduced esophageal dose plan compared to the 11‐beam clinical plan. The mean reduction in V50, V55, V60, V65, and V70 was 2.8, 4.1, 5.9, 7.3, and 9.5 cm^3^, respectively (Fig. 2(a)). The mean reduction in LE50, LE55, LE60, LE65, and LE70 was 2.0, 3.0, 3.8, 4.0, and 4.6 cm, respectively (Fig. 2(b)).

**Figure 3:**
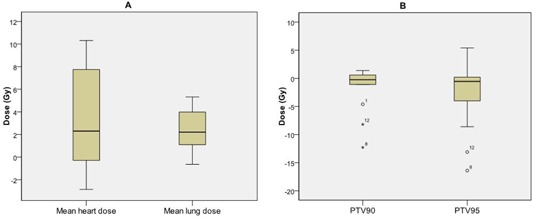
Box plot of change in critical structure doses and PTV coverage between the 11‐beam clinical and reduced esophageal dose plans for all 14 patients: changes in critical structure doses (a) and in 90% and 95% planning target volume (PTV) dose coverage (b) between reduced esophagus dose plans and the 11‐beam clinical plans. Boxes= the 75th and 25th percentile; ′horizontal line= the mean value; whiskers= the range of data values; circles and stars=outliers.

The dose to critical structures was also examined as a change from the 11‐beam clinical plan to the esophagus plan. The mean heart dose decreased 3.0 Gy and the mean lung dose increased 2.4 Gy (Fig. 3(a)). The change in spinal cord V50 was negligible for all patients.

Treatment plan quality was reduced for the esophageal sparing plan compared to the 11‐beam clinical plan for eight patients. The mean decrease in 90% and 95% PTV coverage was 1.7 and 2.8 Gy, respectively (Fig. 3(b)). Normalization of these eight patients’ esophagus plans to recover the lost 95% PTV coverage was computed.

**Figure 4:**
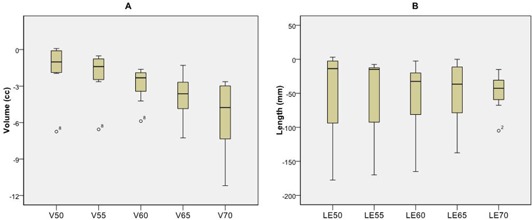
Box plot of change in dosimetric parameters after renormalization between the 11‐beam clinical and reduced esophageal dose plans for the eight patients with reduction in PTV coverage: change in esophageal volume (V; (a)), and length (LE; (b)) receiving doses of 50, 55, 60, 65, 70 Gy between the 11‐beam clinical plans and the reduced esophagus dose plans for patients whose beam outputs were increased to recover the 95% planning target volume of the clinical plan. Boxes= the 75th and 25th percentile; horizontal line= the mean value; whiskers= the range of data values; circles=outliers.

**Figure 5:**
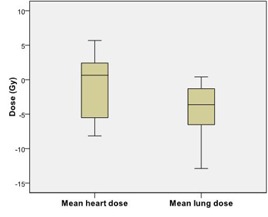
Box plot of change in critical structure doses after renormalization between the 11‐beam clinical and reduced esophageal dose plans for the eight patients with reduction in PTV coverage. Beam outputs were increased to recover the 95% planning target volume of the clinical plan. Boxes= the 75th and 25th percentile; horizontal line= the mean value; whiskers= the range of data values.

After normalization, the esophageal dose values still greatly declined. The V50, V55, V60, V65, and V70 had mean reductions of 1.6, 2.0, 2.9, 3.9, and 5.5 cm^3^, respectively (Fig. 4(a)). The LE50, LE55, LE60, LE65, LE70 Gy had mean reductions of 4.9, 5.2, 5.4, 4.9, and 4.8 cm, respectively (Fig. 4(b)). The critical structure doses increased slightly from their pre‐normalization levels. For the normalized esophagus plans, the mean heart dose decreased 0.9 Gy and the mean lung dose increased 4.4 Gy, compared with the clinical plans (Fig. 5). The spinal cord V50 remained negligible for all eight patients. All patients met the critical structure constraints.

The maximum esophagus dose with interfractional motion simulated was reduced by 1.7 Gy and 3.0 Gy for the clinical plans with 3 mm and 5 mm standard deviations, and 0.5 Gy and 1.4 Gy for the esophagus plans with 3 mm and 5 mm standard deviations, respectively. The maximum dose to the esophagus was 83 and 78 Gy for the clinical plan and the esophagus plan, respectively. The mean LE60 with interfractional motion simulated was reduced by 1 mm for the clinical plan and 5 mm for the esophagus plan. LE60 was 7.8 cm for the 11‐beam clinical plan and 5.0 cm for the esophagus plan. In plans with interfractional motion simulated, the LE60 was distributed abnormally, with a long tail favoring the shorter lengths.

## IV. DISCUSSION

We have studied the difference in using a reduced esophageal dose treatment plan compared to a conventional 11‐beam clinical plan by generating plans for 14 patients with NSCLC using mdaccAutoPlan radiotherapy treatment plan generation algorithm. Treatment planning to emphasize esophageal dose reduction can significantly decrease the volume and length of esophagus that receives high radiation doses. However, in many cases there is some modest reduction in PTV coverage for the reduced esophageal dose plans. Our results show that esophageal reduced dose treatment plans can be normalized to restore 95% PTV coverage, as well as achieve large reductions in the volume and length of esophagus exposed. When restoring PTV coverage, critical structure dose constraints can see an increase, but this increase is minimal on average. These results show reduced esophageal dose plans can be utilized as an alternative to conventional 11‐beam IMRT plans.

Interestingly, the interfractional motion reduced the maximum esophageal dose for both the clinical and the esophagus plan types. The similar reductions of dosimetric values under simulated interfractional motion indicate that the esophagus plans suffered no loss of robustness when compared to the standard plans. This indicates the esophagus receives a better dose distribution than the original treatment plan predicted for these plans. In addition, the influence of interfractional motion on these mdaccAutoPlan‐generated treatment plans does not show discrepancies between the 11‐beam clinical plan and the esophagus plan. Furthermore, the reduced esophagus dose due to simulated motion would seem to imply motion is advantageous and beneficial in terms of esophagus sparing and should be included when modeling normal tissue toxicity in the esophagus.

Our study had a few possible limitations. We examined only the volume and length of esophagus receiving specific doses as dosimetric quantities. Werner‐Wasik et al.^(7)^ showed many metrics may also correlate with esophageal toxicity. Of the 11 studies reviewed in the Werner‐Wasik study, all but two showed significant univariate correlation for volume of receiving greater than a specific dose and grade 2 or higher esophagitis. Length irradiated was also correlated with grade 2 or higher esophagitis for Maguire et al., Ahn et al., and Belderbos et al.^8^, ^9^, ^11^ Both of these dosimetric quantities were reduced in all the esophagus plans.

In addition, tumor size and location were not discriminated in our study. However, all our patients’ tumors were located in the upper half of the esophagus. Krafft et al.^(24)^ showed that the upper portion of the esophagus is more susceptible to acute toxicity. Therefore, the patients examined in this study are representative of those who would exhibit esophageal normal tissue complications.

## V. CONCLUSIONS

Many IMRT treatment plans for lung cancer can accomplish esophageal dose reduction while maintaining adequate PTV coverage, although there is variability among patients. The mdaccAutoPlan algorithm can create alternative reduced esophageal dose plan for NSCLC patients. This provides the clinician an alternative plan where esophageal toxicity is of great concern. In addition, interfractional motion does not adversely affect the quality of reduced esophagus dose plans, and actually shows reduced dose to the esophagus.

## ACKNOWLEDGMENTS

This project was supported in part by NIH/NCI P01 CA021239. The content is solely the responsibility of the authors and does not necessarily represent the official views of the NCI or the NIH. In addition, the work in this study was supported by author's institution start up funds (L. Court).
